# Midnolin Regulates Liver Cancer Cell Growth In Vitro and In Vivo

**DOI:** 10.3390/cancers14061421

**Published:** 2022-03-10

**Authors:** Soo-Mi Kweon, Gayeoun Kim, Yunseong Jeong, Wendong Huang, Ju-Seog Lee, Keane K. Y. Lai

**Affiliations:** 1 Department of Molecular Medicine, Beckman Research Institute of City of Hope, Duarte, CA 91010, USA; skweon@coh.org (S.-M.K.); gakim@coh.org (G.K.); 2 Department of Systems Biology, The University of Texas MD Anderson Cancer Center, Houston, TX 77030, USA; ysjeong@mdanderson.org (Y.J.); jlee@mdanderson.org (J.-S.L.); 3 Department of Diabetes Complications and Metabolism, Beckman Research Institute of City of Hope, Duarte, CA 91010, USA; whuang@coh.org; 4 City of Hope Comprehensive Cancer Center, Duarte, CA 91010, USA

**Keywords:** midnolin, hepatocellular carcinoma, liver cancer, liver carcinogenesis

## Abstract

**Simple Summary:**

Liver cancer is one of the deadliest cancers worldwide. Discovery of novel genes that contribute to the development of liver cancer will provide new insights for better understanding and treating liver cancer. To this end, we recently discovered that expression of the gene midnolin promotes liver cancer and correlates with poor prognosis in liver cancer patients. Targeting midnolin may be useful in future therapy for liver cancer.

**Abstract:**

Hepatocellular carcinoma (HCC) ranks worldwide as one of the most lethal cancers. In spite of the vast existing knowledge about HCC, the pathogenesis of HCC is not completely understood. Discovery of novel genes that contribute to HCC pathogenesis will provide new insights for better understanding and treating HCC. The relatively obscure gene midnolin has been studied for over two decades; however, its biological roles are largely unknown. Our study is the first to demonstrate the functional significance of midnolin in HCC/cancer: Midnolin expression correlates with poor prognosis in HCC patients, and suppression of midnolin severely inhibits tumorigenicity of HCC cells in vitro and in mice and disrupts retinoic acid/lipid metabolism in these cells.

## 1. Introduction

Primary liver cancer ranks worldwide as the second most common cause of cancer death and seventh most commonly occurring cancer [1]. Hepatocellular carcinoma (HCC) comprises ~75% of primary liver cancer [1]. The multifactorial molecular pathogenesis of HCC contributes to its poor response to conventional chemotherapy [2] and dismal prognosis for patients [3]. Moreover, metabolic reprogramming of HCC cells, such as alterations in glucose metabolism (e.g., up-regulation of glycolysis), lipid metabolism (e.g., up-regulation of lipid synthesis and desaturation), and glutamine metabolism (e.g., up-regulation of glutamine synthesis), can drive tumor proliferation and/or drug resistance, thereby also contributing to extremely poor patient prognosis and response to chemotherapy [4,5]. Despite such extensive knowledge about HCC, the pathogenesis of HCC remains to be fully understood. Uncovering novel genes that promote HCC should yield greater understanding of and improved clinical treatments for HCC.

We have previously observed, in novel p300 S89A knock-in mice that displayed enhanced Wnt/CBP/beta-catenin signaling [6], that major metabolic defects existed and midnolin was one of the most significantly differentially expressed genes in livers [7], pointing to a role for midnolin in liver disease. Midnolin, named based on its pattern of expression in mouse embryo midbrain and localization to the nucleolus, was first identified as a potential regulator of neurogenesis-related genes more than 20 years ago by Tsukahara et al. [8]. In addition to having been reported to be highly expressed in E12.5 mouse midbrain, midnolin was reported to be variably expressed in adult mouse heart, brain, spleen, lung, liver, skeletal muscle, kidney, and testis [8]. Initially, midnolin was reported to be localized in the nucleolus and nucleus but not cytoplasm and was purported to contain a C-terminal nucleolar localization signal [8]. Subsequently, midnolin has been reported to be localized in the nucleus (but not nucleolus) and cytoplasm in pancreatic beta cells [9], and in the nucleus and intracellular membranes and possibly in the cytoplasm of PC12 cells [10]. Besides its potential role as a regulator of neurogenesis, midnolin has been reported to potentially serve as: a transcription factor regulating development via control of mRNA transport [11]; a binding partner and negative regulator, via its ubiquitin-like domain, of glucokinase enzyme in pancreatic beta cells [9]; and a regulator of parkin expression and a marker associated with Parkinson’s disease [10]. Midnolin expression has been reported to be induced by NGF (via ERK1/2 and ERK5) and cAMP signaling [10].

Although midnolin has been studied for over two decades, its biological roles, especially in liver and cancer, are largely unknown. Therefore, we investigated the function of midnolin in HCC. Our study is the first to demonstrate the functional significance of midnolin in HCC/cancer: Midnolin expression correlates with poor prognosis in HCC patients, and suppression of midnolin severely inhibits tumorigenicity of HCC cells in vitro and in mice and disrupts retinoic acid/lipid metabolism in these cells.

## 2. Materials and Methods

### 2.1. Cell Culture and Lentiviral Transduction of shRNAs

Mouse hepatocellular carcinoma cell line Hepa1-6 cells were purchased from ATCC (Manassas, VA, USA). Cells were grown and cultivated in Dulbecco’s Modified Eagle Medium (DMEM) with 10% fetal bovine serum (FBS) and 1% penicillin/streptomycin unless otherwise indicated. Cells were maintained in a humidified incubator at 37 °C with 5% CO_2_. Mouse midnolin shRNA plasmids (TL509212), mouse midnolin Open Reading Frame (ORF) clone (MR208176L4), and human midnolin ORF clone (RC210528L4) were purchased from OriGene Technologies (Rockville, MD, USA). Lentiviral particles were prepared by Gene Editing and Viral Vector Core at City of Hope (Duarte, CA, USA) and were used to transduce cells. At 48 h after transduction, cells were replenished with fresh culture medium including puromycin and selected for 7–10 days with change of fresh medium at regular intervals. For isolation of single-cell-derived colony from puromycin selected cells, a limiting dilution of cells was performed to seed into 96-well plate. Once colony formation was observed under microscope, each colony of cells was transferred into larger well size plate and expanded to produce cell stocks that were frozen and could be used in further experiments. mRNA expression of midnolin in each colony was evaluated by RT-qPCR.

### 2.2. Reverse Transcription Quantitative Polymerase Chain Reaction (RT-qPCR)

Total mRNA from cells was extracted by using TRIzol reagent according to the manufacturer’s protocol (ThermoFisher Scientific; San Diego, CA, USA). cDNAs were synthesized by using qScript cDNA Synthesis Kit (Quantabio; Beverly, MA) and used as templates for qPCR with SYBR Green detection method. The sequences of each qPCR primer used in this study were as follows: midnolin (mouse) forward 5′-GTTGTCCCAACGCCTCAAAG-3′, reverse 5′-CAAGGCTTGCATAACGGACTG-3′; midnolin (human) forward 5′-AGAAACGGCTCCGTAGAAAGG-3′, reverse 5′-GACTTGATGTCAGGGTTGACTTC-3′; Gapdh forward 5′-GGTGCTGAGTATGTCGTGGA-3′, reverse 5′-ACAGTCTTCTGGGTGGCAGT-3′; Acsl1 forward 5′-TCCATGCAGTCAGTGGAAATAG-3′, reverse 5′-TTGGCTTCCGAGAACCTAAAC-3′; Aldh1a1 forward 5′-GGAATACCGTGGTTGTCAAGCC-3′, reverse 5′-CCAGGGACAATGTTTACCACGC-3′; Lpl forward 5′-CGGTAACGGGAATGTATGAGAG-3, reverse 5′-GCCAGCTGACACTGGATAAT-3; Lrp1 forward 5′-CGAGAGCCTTTGTGCTGGATGA-3′, reverse 5′-CGGATGTCCTTCTCAATGAGGG-3′; Rbp1 forward 5′-GGATGGTGACAAACTCCAGTGTG-3′, reverse 5′-CAGATCACACCCTCAGCTCTCA-3′; Stra6 forward 5′-GCTGTCTTTGTGGTCCTCTT-3′, reverse 5′-AGGGTAATAGAGGGCTGGATAG-3′; and Ttr forward 5′-CTCGCTGGACTGGTATTTGT-3′, reverse 5′-AGGATCCCTCAGAGGTCTTT-3′.

### 2.3. Cell Proliferation Assay and Colony Formation Assay

For cell proliferation assay, 1.5 × 10^5^ cells were seeded per well of 6-well plate in triplicate. At each time point, cells were trypsinized, and the number of viable cells was counted by automated cell counter Bio-Rad TC20 (Hercules, CA, USA). For colony formation assay, 500–1000 cells were seeded in 6-well plate in triplicate and grown for 10–14 days with cell culture medium refreshed every 2–3 days. On the last day, cells were fixed with 100% methanol for 20 min, washed with water, and incubated with crystal violet solution (0.5% crystal violet in 25% methanol, *w*/*v*) for 5 min at room temperature. Excessive crystal violet solution was rinsed off with water, and stained cells were dried overnight. Entire image of stained cells was visualized by ChemiDoc imaging system (Bio-Rad; Hercules, CA, USA), and cells were observed and colonies counted with bright field microscope.

### 2.4. Orthotopic Transplantation of Hepa1-6 Cells into Mice

All animal experiments were approved by the Institutional Animal Care and Use Committee (IACUC) at City of Hope (Duarte, CA, USA). Immune-competent C57BL/6J male mice were purchased from The Jackson Laboratory (Bar Harbor, ME, USA) and used for experiments at age 6–8 weeks. Mice were randomly assigned into experimental groups. On the day of orthotopic transplantation, 2 × 10^6^ Hepa1-6 midnolin knockdown cells (sh1) or scramble (scr) control cells were prepared in 50 uL of ice-cold PBS per mouse. Then, under anesthesia, each mouse was injected with sh1 cells or scr control cells into the left lobe of liver. At 6 weeks after injection, mice were euthanized and liver tissues were collected and processed for H&E staining. H&E-stained slides were scanned, and selected representative areas were visualized and captured with NDP.view2 software (U12388-01).

### 2.5. RNA Sequencing and Analysis

For RNA sequencing (RNA-seq), total RNA from cells was extracted using Quick RNA miniprep kit from Zymo Research (Irvine, CA, USA) and sent to Integrative Genomics Core at City of Hope (Duarte, CA, USA) for subsequent preparation of an RNA-seq library, sequencing, and analysis. Briefly, RNA-seq libraries were prepared with Kapa RNA HyperPrep kit with RiboEase (Kapa Biosystems, Wilmington, MA, USA, Cat KR1351) according to the manufacturer’s protocol. All library samples were validated with the Agilent Bioanalyzer, quantified with Qubit, and sequenced on Illumina HiSeq 2500 (San Diego, CA, USA) with single-read mode/50 million depths. For identifying differentially expressed genes (DEGs) based on RPKM (Reads Per Kilobase of transcript, per Million mapped reads), the RNA-seq reads were aligned to mm10 genome assembly using Tophat2 (v2.0.8) with default settings. Then, the gene expression levels were counted to obtain raw counts with HTSeq (v0.11.2) against RefSeq Genome Reference Consortium Mouse Build 38 annotation. The count data were normalized using the trimmed mean of M values (TMM) method, implemented in the Bioconductor package edgeR (v.3.30.3) to obtain the normalized RPKM value. Genes were considered differentially expressed between knockdown (sh1 and sh2) samples and scramble (scr) control samples if absolute fold change was ≥1.5, FDR < 0.05, and at least one sample had RPKM > 1. Then, the number of up-regulated DEGs was further stringently reduced using the criterion of RPKM ≥ 20 for the mean of all knockdown (sh1 and sh2) samples, whereas the number of down-regulated DEGs was further stringently reduced using the criterion of RPKM ≥ 20 for the mean of all scr control samples. To create heatmaps with selected genes of interest, the values of RPKM in all samples for each gene were visualized using percent scale.

### 2.6. Bioinformatics Analysis of Data from Hepatocellular Carcinoma Patient Cohorts

Transcriptomic and clinical data from primary hepatocellular carcinoma (HCC) were obtained from Gene Expression Omnibus database (GSE16757 for Korea, GSE54236 for Modena, and GSE22058 for University of Hong Kong (UHK) [12,13,14]). RNA sequencing and clinical data from The Cancer Genome Atlas Liver Hepatocellular Carcinoma (TCGA-LIHC) cohort were obtained from The Human Protein Atlas (https://www.proteinatlas.org/ENSG00000167470-MIDN/pathology/liver+cancer) (accessed on 22 May 2021). Whereas the TCGA cohort was dichotomized by median value of midnolin expression, the rest of the other cohorts (Korea, Modena, and UHK) were dichotomized by 40th percentile cutoff.

### 2.7. Statistical Analysis

Numerical data were expressed as means ± SD. Statistical significance of difference was assessed by the Student’s *t*-test (two-tailed). *p* < 0.05 was considered statistically significant.

## 3. Results

### 3.1. Suppression of Midnolin Reduces Tumorigenicity of Liver Cancer Cells

**Figure 1:**
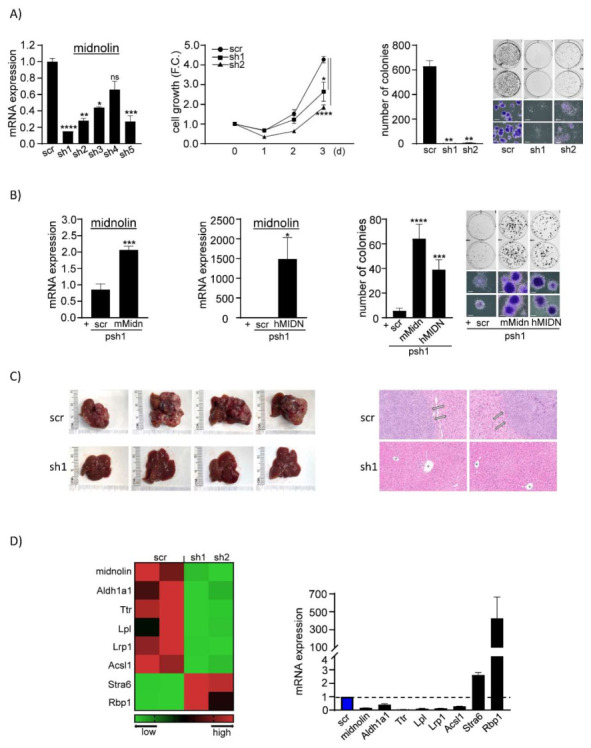
Midnolin expression is correlated with cell proliferation and tumor formation. (**A**) **Left**, mRNA expression of midnolin in clones of Hepa1-6 cells with stable knockdown of midnolin (sh1 to sh5) versus stable expression of scramble (scr) control, which were generated by lentivirus transduction of shRNA versus scr control, respectively (two-tailed *t*-test, each knockdown clone versus scr control clone, *n* = 3). **Center**, Cell proliferation assay with Hepa1-6 midnolin knockdown cells (sh1 and sh2) versus scr control cells for 3 days total (two-tailed *t*-test, each knockdown clone versus scr control clone, *n* = 3). F.C. = fold change. **Right**, Colony formation assay with Hepa1-6 midnolin knockdown cells (sh1 and sh2) versus scr control cells (two-tailed *t*-test, each knockdown clone versus scr control clone, *n* = 3). Cells were stained with crystal violet and visualized with ChemiDoc and light microscope (4× magnification, scale bar = 500 um). (**B**) Rescue of Hepa1-6 midnolin knockdown cells (sh1) by exogenous expression of mouse midnolin (mMidn) or human midnolin (hMIDN) via lentivirus transduction. **Left and Center**, mRNA expression of midnolin in Hepa1-6 midnolin knockdown cells (sh1), which were transduced with lentivirus expressing mMidn, hMIDN, or scr control to generate respective groups of pooled cells, i.e., psh1 + mMidn, psh1+hMIDN, psh1 + scr (two-tailed *t*-test, psh1 + mMidn or psh1 + hMIDN versus control psh1 + scr, *n* = 3). **Right**, Colony formation assay with each group of rescued cells versus control cells (two-tailed *t*-test, psh1 + mMidn or psh1 + hMIDN versus control psh1 + scr, *n* = 3). Cells were stained with crystal violet and visualized with ChemiDoc and light microscope (4× magnification, scale bar = 500 um). (**C**) Orthotopic transplantation of Hepa1-6 midnolin knockdown cells (sh1) versus scr control cells into the livers of immune-competent C57B/6J mice. **Left**, Gross examination reveals tumor formation in the livers of mice orthotopically transplanted with Hepa1-6 scr control cells versus no tumor formation in the livers transplanted with midnolin knockdown cells (sh1). **Right**, Representative images of H&E staining of liver tissues from mice orthotopically transplanted with Hepa1-6 midnolin knockdown cells (sh1) versus scr control cells. Histologic sections from livers transplanted with scr control cells show involvement by hepatocellular carcinoma: The tumor grows in sheets (block arrows) and is characterized by epithelioid cells with vesicular nuclei, irregular nuclear contours, occasional prominent nucleoli, containing scant-to-moderate amphophilic cytoplasm, and exhibiting an increased nucleus:cytoplasm ratio and increased cellular density, whereas the background, non-neoplastic hepatocytes contain regular, round nuclei and abundant eosinophilic cytoplasm. Histologic sections from livers transplanted with knockdown cells (sh1) show normal hepatic parenchyma with retained lobular architecture and scattered portal tracts (*) harboring their usual structures, including portal veins and interlobular bile ducts, with no evidence of involvement by malignancy. H&E-stained slides were scanned, and selected representative areas were visualized and captured with NDP.view2 software (U12388-01) (20× magnification, scale bar = 100 um). (**D**) Analysis of differentially expressed genes (DEGs) by RNA-seq and RT-qPCR in Hepa1-6 midnolin knockdown cells (sh1 and sh2) versus scr control cells. **Left**, Partial heatmap derived from RNA-seq analysis of sh1 and sh2 versus scr control cells. **Right**, Validation of RNA-seq data by RT-qPCR with specific pairs of primers for each gene of interest. mRNA expression of each gene of interest in sh1 versus scr control cells (with scr control cell mRNA expression designated as 1 (=100%) as indicated by dotted line). Representative data from 3 independent experiments (*n* = 3). Numerical data were expressed as means ± SD. * *p* < 0.05, ** *p* < 0.01, *** *p* < 0.001, **** *p* < 0.0001, and ns (not significant).

To investigate the role of midnolin in liver cancer cells, we generated clones of Hepa1-6 cells with stable knockdown of midnolin (sh1 to sh5) versus stable expression of scramble (scr) control, via lentivirus transduction followed by selection of single cells by limiting dilution. Knockdown clones sh1 to sh5 showed suppression of midnolin ranging from 34% to 85% versus scr control clone, as assessed by RT-qPCR (Figure 1A, left). Given that the parameters of cell growth and colony formation are known to be correlated with tumorigenicity, we chose clones sh1 and sh2, which exhibited strong suppression of midnolin, to assess the effect of such suppression on these parameters as a readout for tumorigenicity. We found that cell growth of sh1 and sh2 was reduced by ~40% and ~60%, respectively, at 3 days post plating of cells versus scr control, as assessed by cell counting (Figure 1A, center). Colony formation was dramatically suppressed with sh1 and sh2 compared to scr control (Figure 1A, right). Hence, we concluded that suppression of midnolin reduces the tumorigenicity of liver cancer cells.

### 3.2. Exogenous Expression of Midnolin Rescues Tumorigenicity of Liver Cancer Cells

To confirm the specificity of midnolin knockdown-mediated suppression of colony formation, we tested whether exogenous expression of mouse midnolin (mMidn) and human midnolin (hMIDN) (which show ~82% identity at the mRNA level and ~84% identity at the protein level) in Hepa1-6 knockdown cells would rescue such suppression of colony formation. To do so, we first transduced sh1 (clone with stable midnolin knockdown) with lentivirus expressing mMidn, hMIDN, or scr control to generate respective groups of pooled cells, i.e., psh1 + mMidn, psh1 + hMIDN, psh1 + scr. After confirming that psh1 + mMidn and psh1 + hMIDN exhibited up-regulation of midnolin (Figure 1B, left and center) compared with control (psh1 + scr), we subjected these different groups of cells to the colony formation assay. We found that exogenous expression of mMidn or hMIDN in knockdown cells rescued suppressed colony formation when compared with control (psh1 + scr) (Figure 1B, right).

### 3.3. Suppression of Midnolin Prevents Liver Tumor Formation in Mice

We next tested the in vivo effect of midnolin knockdown on liver tumorigenesis. Immune-competent C57BL/6J mice were orthotopically transplanted with sh1 (stable midnolin knockdown) or scr control cells and euthanized 6 weeks later. All mice with scr control transplantation developed conspicuous liver tumors, whereas no mice transplanted with sh1 had visible tumors (Figure 1C, left and Figure S1). Histologic sections from livers transplanted with scr control showed obvious involvement by HCC, whereas those from livers transplanted with sh1 showed no evidence of involvement by malignancy (Figure 1C, right). These in vivo results are consistent with our in vitro results, which showed that suppression of midnolin inhibits, whereas rescue of midnolin restores, colony formation/tumorigenicity, demonstrating the importance of midnolin in liver tumorigenesis in vivo.

### 3.4. Midnolin Knockdown-Mediated Disruption of Retinoic Acid/Lipid Metabolism

To gain insight into the mechanism by which midnolin regulates liver tumorigenesis, we performed RNA-seq on sh1 and sh2 (with stable midnolin knockdown) versus scr control. Initial analysis of RNA-seq data showed that a total of 1327 genes (703 genes up-regulated, 624 genes down-regulated) were differentially expressed in knockdown cells (sh1 and sh2) compared to scr control, using absolute 1.5-fold cutoff (FDR < 0.05) (Table S1). To arrive at a more robust set of DEGs, the number of up-regulated DEGs was further stringently reduced using the criterion of RPKM ≥20 for the mean of all knockdown samples, whereas the number of down-regulated DEGs was further stringently reduced using the criterion of RPKM ≥20 for the mean of all scr control samples. Thus, the number of DEGs was reduced to a total of 489 genes (234 genes up-regulated, 255 genes down-regulated; see Table S1), and IPA canonical pathway analysis was subsequently performed on this set of DEGs (Figure S2). We found that some of the most highly differentially expressed genes are involved in retinoic acid metabolism or lipid metabolism (Figure 1D, left). Among the most down-regulated genes in midnolin knockdown cells, as confirmed by RT-qPCR (Figure 1D, right), were Aldh1a1 (−60%) which converts retinaldehyde (derived from retinol) to retinoic acid [15], and Ttr (−96%) which transports retinol in blood [16]. In stark contrast to this observed insufficiency in retinoid signaling, we found that Stra6, which transports retinol across the cell membrane and transfers retinol to cellular retinol-binding protein Rbp1 [16], as well as Rbp1, which delivers retinol to downstream metabolizing enzyme [16], were among the most up-regulated genes (1.6-fold and 425-fold, respectively) in midnolin knockdown cells. We hypothesize that such up-regulation may be an upstream compensatory response to the observed insufficiency in retinoid signaling. Furthermore, hepatic Lpl, which supports clearance of blood triglyceride [17], Lrp1, which participates in uptake by liver of chylomicrons and very low-density lipoproteins from blood [18], and Acsl1, which activates long-chain fatty acids and facilitates lipid biosynthesis [19], were highly down-regulated (−90%, −87%, and −70%, respectively) in midnolin knockdown cells. Indeed, dysregulation of Aldh1a1 [20], Ttr [21], Rbp1 [22], Lpl [23], Lrp1 [24], and Acsl1 [25] in HCC has previously been reported. Given the important role of retinoic acid metabolism [26] and that of lipid metabolism [27] in regulating cell growth/transient amplification of cells and differentiation in cancer, we propose that midnolin knockdown-mediated disruption of such metabolic pathways may explain the observed reduction in tumorigenic potential of midnolin knockdown cells in vitro and in mice.

### 3.5. Midnolin Expression Correlates with Poor Prognosis in HCC Patients

**Figure 2:**
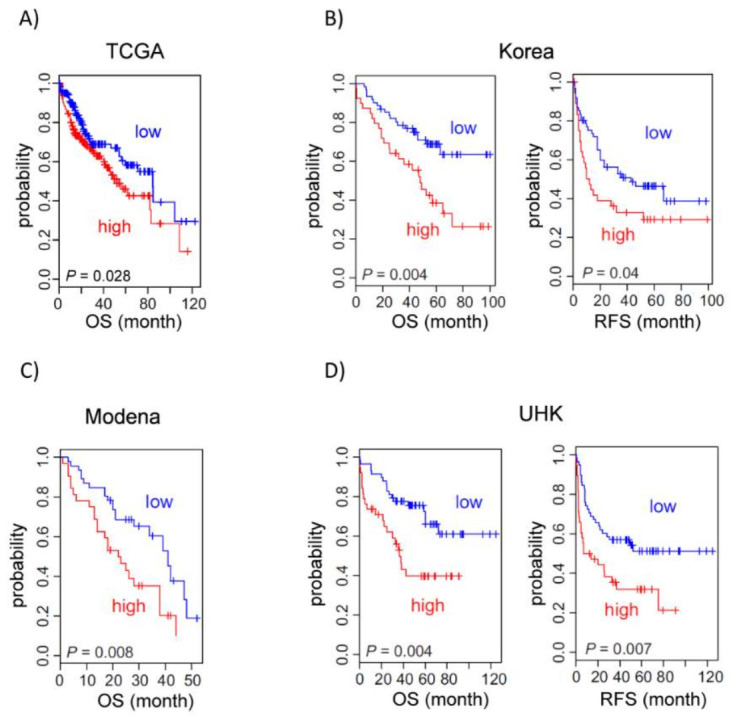
Kaplan–Meier survival curves show that midnolin expression in hepatocellular carcinoma (HCC) correlates with poor patient prognosis. When patients were dichotomized by midnolin expression level in HCC, high expression of midnolin was significantly associated with poor overall survival (OS) and poor relapse-free survival (RFS). (**A**) TCGA data (*n* = 365, median cutoff); (**B**) Korea cohort (*n* = 100, 40th percentile cutoff) [12]; (**C**) Modena cohort (*n* = 76, 40th percentile cutoff) [13]; and (**D**) University of Hong Kong (UHK) cohort (*n* = 96, 40th percentile cutoff) [14].

In addition, we assessed the clinical relevance of midnolin expression in four independent HCC patient cohorts (Korea, Modena, University of Hong Kong (UHK), and The Cancer Genome Atlas (TCGA)) [12,13,14] (Figure 2). When patients were dichotomized by midnolin expression level, we found that high expression of midnolin is significantly associated with poor overall survival. In good agreement with this, high midnolin expression is also significantly associated with poor relapse-free survival in two HCC patient cohorts. Additional analysis of midnolin expression in HCC tumors, surrounding non-tumor liver tissues, and normal liver in the National Cancer Institute cohort [28,29] showed that midnolin expression is highest in HCC tumors and lowest in normal liver (Figure S3), further suggesting that midnolin expression is associated with HCC.

## 4. Discussion

Midnolin has previously been reported to potentially regulate neurogenesis-related genes [8] and pancreatic beta cell glucokinase activity [9], and possibly be associated with Parkinson’s disease though the data are controversial [10,30,31]. Otherwise, very little is known about midnolin. Using a combination of in vitro/in vivo models and RNA-seq, and identifying midnolin’s significant clinical relevance in HCC, our study is the first to demonstrate a functional role for midnolin in cancer. We found that midnolin expression correlates with poor prognosis in HCC patients, and suppression of midnolin severely inhibits growth of HCC cells in vitro and in mice and disrupts retinoic acid/lipid metabolism in these cells. Further studies are necessary to detail the mechanism by which midnolin metabolically regulates hepatocarcinogenesis. Additionally, given that midnolin expression seems to affect overall survival more in the Asian cohorts (versus Modena cohort) that we analyzed, it is tempting to speculate that midnolin may play more of a role in hepatitis B virus (HBV)-mediated tumorigenesis since HBV infection is the dominant etiology in Asian cohorts. Further studies are needed to clarify this concept.

## 5. Conclusions

Although midnolin has been studied for over two decades, its biological roles are largely unknown. Our study is the first to demonstrate the functional significance of midnolin in HCC/cancer: Midnolin expression correlates with poor prognosis in HCC patients, and suppression of midnolin severely inhibits tumorigenicity of HCC cells in vitro and in mice and disrupts retinoic acid/lipid metabolism in these cells. Targeting midnolin and associated cancer metabolism may be useful in future therapy for HCC.

## Data Availability

RNA-seq data will be made publicly available via NCBI Gene Expression Omnibus (GEO) (accession number GSE197620). Study materials will be made available to other researchers from the corresponding author on reasonable request.

## Supplementary Materials

The following supporting information can be downloaded at: https://www.mdpi.com/article/10.3390/cancers14061421/s1, Figure S1: Analysis of tumor formation by orthotopic transplantation with scr versus sh1 cells; Figure S2: Analysis of gene expression by midnolin knockdown; Figure S3: Expression of midnolin in HCC tumors, surrounding non-tumor liver tissues, and normal liver; Table S1: List of Differentially Expressed Genes.
