# Supplementary material for: Midnolin Regulates Liver Cancer Cell Growth In Vitro and In Vivo
Source: Cancers (Basel). 2022 Mar 10;14(6):1421. doi: 10.3390/cancers14061421 (PMC8946164; doi:10.3390/cancers14061421)
Supplement: Supplementary file 1 [file cancers-14-01421-s001.zip › cancers-1555700-supplementary.pdf]

Supplemental Materials

Supplemental Methods

Bioinformatics Analysis of Data from NCI cohort
Expression of midnolin from liver tissues was extracted from National Cancer Institute (NCI) cohort from previous studies [1,2]. Data from NCI cohort include gene expression of HCC tumors, matched surrounding non-tumor tissues, and normal liver from healthy individuals. Gene expression data were normalized to normal liver (log2 ratio).

References

1. Lee, J.S.; Chu, I.S.; Heo, J.; Calvisi, D.F.; Sun, Z.; Roskams, T.; Durnez, A.; Demetris, A.J.; Thorgeirsson, S.S. Classification and prediction of survival in hepatocellular carcinoma by gene expression profiling. *Hepatology* **2004**, *40*, 667-676, doi:10.1002/hep.20375.
2. Lee, J.S.; Heo, J.; Libbrecht, L.; Chu, I.S.; Kaposi-Novak, P.; Calvisi, D.F.; Mikaelyan, A.; Roberts, L.R.; Demetris, A.J.; Sun, Z.; et al. A novel prognostic subtype of human hepatocellular carcinoma derived from hepatic progenitor cells. *Nat Med* **2006**, *12*, 410-416, doi:10.1038/nm1377.

Supplemental Figures

Treatment	Animal ID #	Body Weight (g)	Liver (with tumor) Weight (g)	Liver Weight (g) to Body Weight (g) ratio (%)	Tumor Weight (g)	Tumor Length (mm)	Tumor Width (mm)	Tumor Volume (mm3)
scr	1	21.51	2.45	11.4	1.33	19.6	8.09	641.4
	2	20.22	3.98	19.7	2.63	24.36	17.98	3937.6
	3	22.04	4.03	18.3	2.55	24.85	15.51	2989.0
	4	22.38	4.10	18.3	2.92	22.46	18.37	3789.6
sh1	5	24.71	1.16	4.7	N.D.	N.A.		
	6	25.65	1.18	4.6				
	7	23.56	1.00	4.2				
	8	26.48	1.23	4.6				

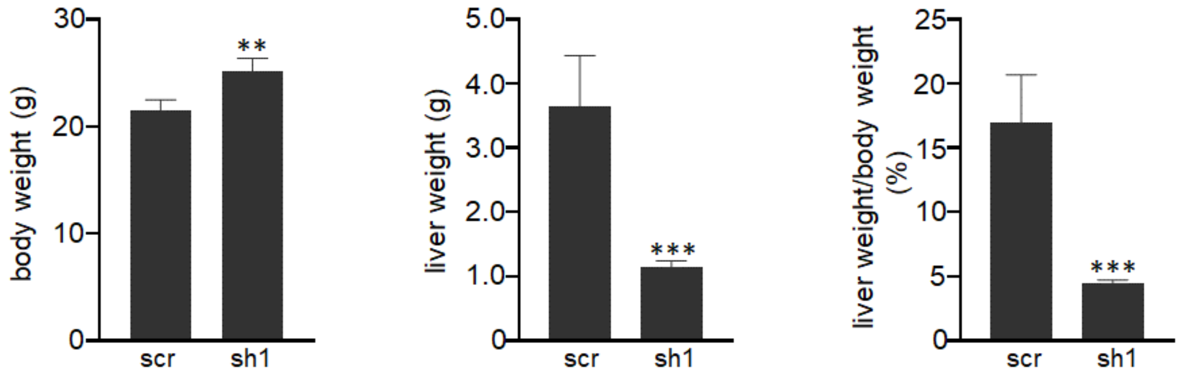

Figure S1. Analysis of tumor formation by orthotopic transplantation with scr versus sh1 cells. Body weight, liver weight, tumor weight and volume (if tumor present), and liver weight to body weight ratio for each mouse were assessed for comparison between groups (n=4/group). Numerical data were expressed as means ± SD. **P<0.01, ***P<0.001. N.D. (Not Determined), N.A. (Not Applicable).

A)

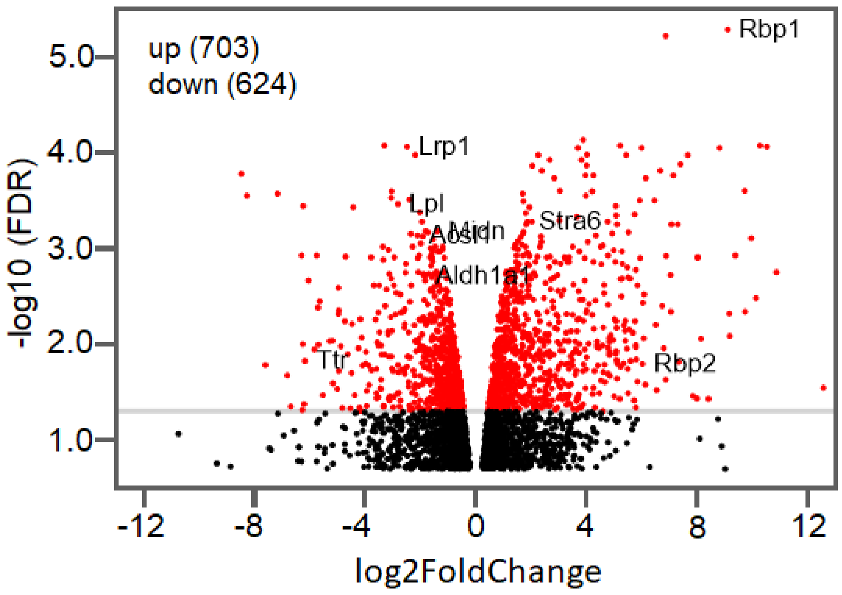

B)

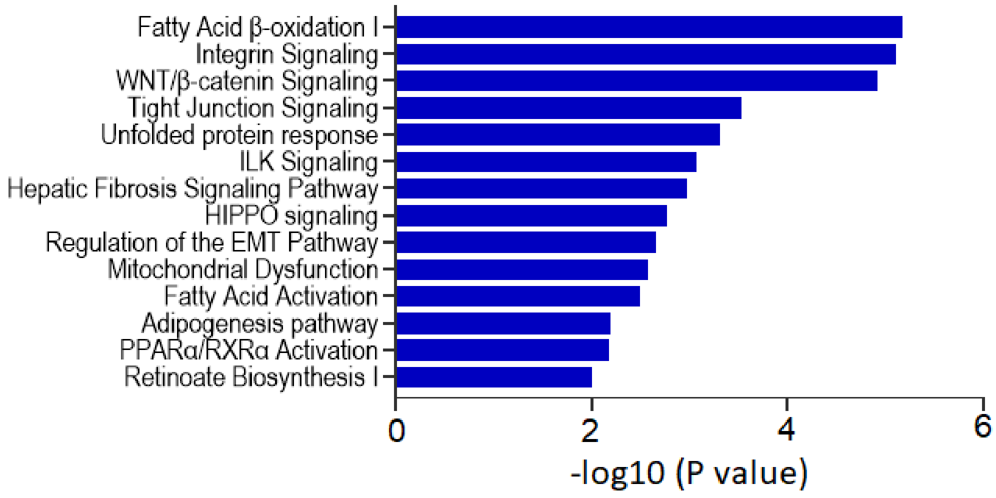

Figure S2. Analysis of gene expression by midnolin knockdown. A) Volcano Plot demonstrating differentially expressed genes (DEGs) in sh1 and sh2 midnolin knockdown cells compared to scr control cells (FDR <0.05, absolute fold change ≥1.5). Total 1327 genes were identified as DEGs (703 of up-regulated genes, 624 of down-regulated genes). B) Canonical Pathway analysis by IPA. Threshold value was designated as 1.3, the default value by IPA.

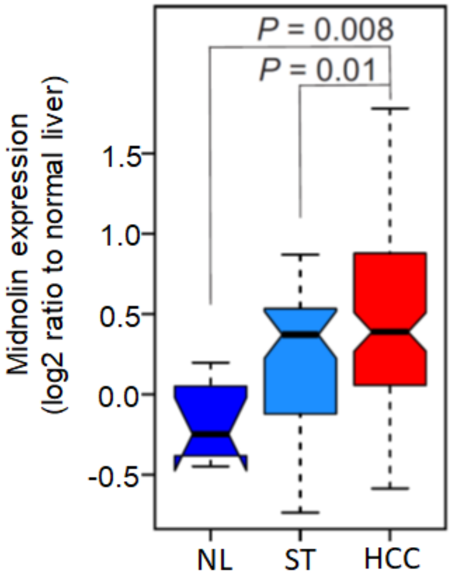

Figure S3. Expression of midnolin in HCC tumors, surrounding non-tumor liver tissues, and normal liver. Midnolin expression is highest in HCC tumors and lowest in normal liver in National Cancer Institute cohort. P-values indicate statistical significance from Student t-test. NL, normal liver; ST, surrounding tissues; HCC, hepatocellular carcinoma.
